# Set Up for Failure: Pre-Existing Autoantibodies in Lung Transplant

**DOI:** 10.3389/fimmu.2021.711102

**Published:** 2021-08-11

**Authors:** Alexander McQuiston, Amir Emtiazjoo, Peggi Angel, Tiago Machuca, Jason Christie, Carl Atkinson

**Affiliations:** ^1^Department of Microbiology and Immunology, Medical University of South Carolina, Charleston, SC, United States; ^2^Division of Pulmonary, Critical Care and Sleep Medicine, University of Florida, Gainesville, FL, United States; ^3^Department of Cell and Molecular Pharmacology and Experimental Therapeutics, Medical University of South Carolina, Charleston, SC, United States; ^4^Department of Surgery, University of Florida, Gainesville, FL, United States; ^5^Department of Medicine, University of Pennsylvania, Philadelphia, PA, United States

**Keywords:** lung transplant, complement, Autoantibodies, primary graft dysfunction, chronic lung allograft dysfunction, glycans

## Abstract

Lung transplant patients have the lowest long-term survival rates compared to other solid organ transplants. The complications after lung transplantation such as primary graft dysfunction (PGD) and ultimately chronic lung allograft dysfunction (CLAD) are the main reasons for this limited survival. In recent years, lung-specific autoantibodies that recognize non-HLA antigens have been hypothesized to contribute to graft injury and have been correlated with PGD, CLAD, and survival. Mounting evidence suggests that autoantibodies can develop during pulmonary disease progression before lung transplant, termed pre-existing autoantibodies, and may participate in allograft injury after transplantation. In this review, we summarize what is known about pulmonary disease autoantibodies, the relationship between pre-existing autoantibodies and lung transplantation, and potential mechanisms through which pre-existing autoantibodies contribute to graft injury and rejection.

## Introduction

Lung transplantation (LTx) is the only viable option for many chronic end-stage pulmonary diseases (CPD). The number of LTx performed annually in the US is rapidly increasing, and the demand for donor lungs far exceeds availability. Despite the improved surgical techniques and post-transplant management, the long-term survival has not significantly improved over the last decade and remains the lowest compared to other solid organ Tx (1). According to the International Society of Heart and Lung Transplantation (ISHLT) registry data, median survival after lung transplantation is 6.5 years, the worst amongst all solid organ transplantation, and is, in large part, the result of chronic allograft dysfunction (CLAD). Primary graft dysfunction (PGD) is a common early complication after LTx, and a major risk factor for development of CLAD. PGD occurs within the first 72 hours after transplantation and factors such as the recipient’s underlying lung disease, donor medical history, recipient/donor interaction, severity of post-Tx complications all play an integral part in determining LTx success.

Arguably, organ transplantation was made possible through optimization of surgical techniques and subsequent manipulation of the immune system. Significant improvements have been made in controlling the recipient’s immune system post-transplant, largely by modulating T cell immune responses. While modulation of adaptive immunity is essential for graft survival other factors occurring earlier in the transplant process also play a role in graft injury. Initial immune-mediated graft injury occurs upon reperfusion, referred to as ischemia reperfusion injury (IRI). Following IRI, activation of innate and adaptive immune response drives targeted graft damage. This damage manifests as post-Tx complications such as PGD, acute rejection, and CLAD, all of which predispose to increased risk of mortality. The generally accepted model of immune-mediated graft damage proposes a feed-forward mechanism starting with donor graft injury, IRI-mediated immune activation and graft damage, thereby promoting subsequent adaptive immune activation and T cell specific graft damage. However, this model does not take into account the recipients’ lung specific pre-Tx immune system. Patients with end-stage lung diseases, such as chronic obstructive pulmonary disease (COPD) and interstitial lung disease (ILD) often have autoimmunity, which is increasingly being recognized as a potential driver of graft injury post transplantation (2, 3).

The presence of a lung-specific autoreactive immune system pre-transplant may well predispose targeted attack of the donor lung upon implantation, and further exacerbate the alloimmune effector mechanisms activated post-transplant. Immune-mediated graft damage remains a major obstacle, and manipulation of the recipient immune system currently only occurs upon transplantation. Therefore, gaining a better understanding of the immune factors present within the LTx recipient prior to transplantation and how these factors contribute to shaping post-transplant graft outcomes can have a significant impact on the patient outcome. In this review, we will focus on the impact of pulmonary disease-associated autoantibodies and pulmonary disease-specific autoantibodies in LTx, and postulate on the clinical significance of autoantibodies identified in patients.

## Pulmonary Diseases and Autoimmunity: Autoantibodies

Over the last 20 years, accumulated data proports a role for autoimmunity in pulmonary disease pathogenesis and progression of certain lung pathologies (4). How pulmonary disease autoimmunity arises and its influence on disease progression is not fully understood; however, it is generally accepted that autoimmune factors, such as autoantibodies and autoreactive T cells, play critical roles in disease perpetuation.

Autoantibodies are antibodies produced by the immune system with reactivity to self-antigens. Multiple mechanisms can render host molecules antigenic, interestingly, those same mechanisms can occur during or as a result of pulmonary disease onset. For example, environmental exposure to pollutants, such as those found in cigarette smoke, a major risk factor for COPD development, can drive mutations and post-translational modifications such as oxidation, carbonylation, and citrullination to pulmonary peptides rendering them antigenic (5). Fibrotic-associated damage, commonly found in pulmonary diseases and directly correlated with ROS-mediated oxidative stress, can lead to the exposure of cryptic neoepitopes that act as pathogenic antigens that drive inflammation and injury (6). Autoantibodies against a wide spectrum of self-antigens have been identified in patients with lung disease and rodent models of disease (7–9). The clinical relevance of autoantibodies identified in lung disease patients is an area of intense debate. While it is clear that a majority of lung disease patients present with autoantibodies, whether autoantibodies contribute to pathogenesis, are useful biomarkers, or simply epiphenomenon of the underlying disease remains to be elucidated. Below, we highlight recent advances in the association between autoantibodies and COPD and ILD patients (**Table 1**).

**Table 1:** Autoantibody titers correlate with disease progression.

Autoantibody	Ig Isotype	Disease	Association with Severity	Citation
HBEC	IgG/IgA	COPD	Most IgG/A Positive patients were GOLD Stage III and IV	(48)
Elastin	IgG	COPD	Decreased with severity (GOLD Stage)	(49)
Elastin	IgG	COPD	Increased with severity	(50)
Cytokeratin 18	IgG/IgA/IgM	COPD	Increased with severity (GOLD Stage); Correlated with FEV1 (L) and FEV1 (%) Predicted	(51, 52)
Cytokeratin 19	IgG/IgA/IgM	COPD	Increased with severity (GOLD Stage)	(51)
CD80	IgG	COPD	Increased with severity (GOLD Stage)	(53)
Carbonyl Modified Proteins	IgG	COPD	Increased with severity (GOLD Stage)	(54)
Serum Albumin	IgG	COPD	Increased with severity (GOLD Stage)	(54)
ANA	IgG	COPD	Increased with severity (GOLD Stage)	(55–57)
ASMA	IgG	COPD	Increased with severity (GOLD Stage)	(55)
Anti-Tissue	IgG	COPD	Increased with severity (GOLD Stage)	(57)
GRP78	IgG	COPD	Increased with severity (GOLD Stage)	(58)
B2-adrenergic receptor	IgG	COPD	Increased with severity (GOLD Stage)	(59)
Ro52	IgG	ILD	Increased with severity	(60)
MDA5	IgG	ILD	Increased with severity	(22)
Cyclic Citrullinated Peptides	IgG	ILD	Increased with severity	(61, 62)
CXCR3	IgG	ILD	Increased with severity	(63)
CXCR4	IgG	ILD	Increased with severity	(63)
Periplakin	IgG	ILD	Increased with severity	(64)

### Autoantibodies in Chronic Obstructive Pulmonary Disease

Chronic obstructive pulmonary disease (COPD) is a group of progressive pulmonary disorders characterized by chronic pulmonary inflammation and damage to small airways and alveolar airspace that results in airflow limitation. The specific pathogenesis of COPD remains unknown, but it is widely accepted that cigarette smoke (CS) exposure is one of the leading causes. CS promotes innate and adaptive immune cell infiltration into the lung and airways of COPD patients (10, 11). Increased immune cell presence and activity in the lungs is hypothesized to promote progressive tissue destruction, a hallmark of COPD and critical in COPD pathogenesis.

Autoantibodies and autoantigens have been successfully identified in COPD patients and animal models of disease. A systematic review of all clinical studies investigating autoantibodies in the context of COPD was published in 2019 by Byrne et al. (12). The review focuses on 42 peer-reviewed articles that investigate one or multiple autoantibodies. The most well studied autoantibodies include anti-endothelial/epithelial cell autoantibodies (anti-AECA), Rheumatoid factor (RF), anti-cytokeratin (anti-CK), anti-nuclear autoantibodies (ANAs), anti-collagen, anti-cyclic citrullinated peptide autoantibodies (anti-CCP), anti-elastin, anti-smooth muscle autoantibodies (ASMA), and anti-neutrophil cytoplasmic autoantibodies (ANCA) (12). All other autoantibodies mentioned (approximately 9) in the review were investigated in less than three studies. Overall, successful identification of multiple different autoantibodies supports the hypothesis that autoimmunity is prevalent in COPD patients, however, much more research is needed to determine the clinical significance of these autoantibodies.

Many of the most well-studied autoantibodies, such as the ones listed above, are common to other autoimmune diseases such as rheumatoid arthritis, systemic sclerosis, and lupus (13). While those autoantibodies may still be clinically relevant in COPD, identifying disease specific autoantibodies may prove to be more useful as biomarkers, and improve diagnoses and treatment. The complex heterogeneity among COPD patients makes it difficult to identify a single disease specific autoantibody/autoantigen common to all patients, therefore, establishing a panel or signature of autoantibodies may be more useful. A recent study analyzed 19,000 human proteins by protein microarray and found that COPD patients had higher autoantibody titers towards extracellular proteins and neutrophil granule proteins compared to controls (14). Interestingly, IgM and IgG autoantibodies against intracellular antigens were lower in COPD patients, potentially because intracellular autoantibodies are associated with maintaining homeostasis (14–16). The fact that some autoantibody titers are increased while others are decreased in COPD patients compared to normal controls further supports the need for autoantibody profiling. Although the aforementioned data provides the start of a global understanding of the autoantibody landscape, multiple gaps in our knowledge remain.

The most glaring gap in the literature, as discussed in Duncan 2012, is the functional characterization of autoantibodies in COPD pathogenesis (17). Thus far, very little is known whether the autoantibodies identified in COPD patients are pathogenic, beneficial, biomarkers, or simply epiphenomena of the underlying disease. The accepted hypothesis is that the autoantibodies identified contribute to COPD pathogenesis by promoting immune activation, targeted tissue destruction, and cell death. How the autoantibodies promote such a response is unknown. There are multiple autoantibody characteristics and mechanisms that could promote different pathogenic processes, as we discuss in detail later in this review.

### Interstitial Lung Disease and Autoantibodies

Interstitial lung disease (ILD) is a group of multiple pulmonary diseases characterized by progressive scarring or fibrosis of the lungs. ILD is an extremely complex disease because it is commonly associated with connective tissue diseases (CTD-ILD) such as rheumatoid arthritis, lupus, and sclerosis, or non-connective tissue disease origin referred to as interstitial pneumonia with autoimmune features (IPAF). In this context of CTD-ILD, ILD can be one of many manifestations of an established CTD or may be the only manifestation of the CTD. Interestingly, many CTDs can be categorized as autoimmune disorders suggesting that ILD may also comprise an autoimmune component. For example, ILD manifests and is the leading cause of mortality in Systemic-Sclerosis, an extensively studied autoimmune disease with anti-nuclear autoantibodies (ANA) (anti-centromere antibodies, anti-topoisomerase I antibodies, and anti-RNA polymerase antibodies) and anti-endothelial cell antibodies (AECA) in greater than 90% of patients with potential pathogenic roles (18). Additionally, ILD occurs in upwards of 80% of patients with myositis, a rare autoimmune disease that presents with myositis-specific autoantibodies (MSAs; anti-synthetase and anti-CADM140/MDA5).

Whether or not the majority of autoantibodies identified in CTD-ILD patients are involved in disease pathogenesis remains unknown, but their clinical significance as diagnostic markers that shape patient is growing in significance. For example, a subset of clinically amyopathic dermatomyositis (CADM) patients with rapidly progressive ILD have been shown to present with circulating anti-CADM140/MDA5 autoantibody (19). Studies have demonstrated that anti-CADM140/MDA5 positive patients are at a higher risk for developing ILD, and are at a higher risk of ILD-related respiratory failure and death (20–23). While it remains unknown if anti-CADM140/MDA5 play a pathogenic role, its presence can dramatically change clinical intervention strategies.

In 2011, guidelines from the American Thoracic Society/European Respiratory Society/Japanese Respiratory Society and Latin American Thoracic Association highlight ANA, anti-CCP, and RF as necessary autoantibodies to test for in all potential ILD patients (24). Not surprisingly, similar to COPD, numerous other autoantibodies have been discovered in ILD patients with a broad range of frequencies, associations, and techniques. An extensive review of autoantibodies in CTD-ILD and IPAF patients and their clinical importance was recently compiled by Jee et al. (25). The review article provides insight from over 100 articles evaluating the clinical relevance of autoantibodies in CTD-ILD and IPAF, demonstrating their utility, while also bringing light to hurdles that limit our understanding. The vast heterogeneity of CTD-ILD and IPAF disease phenotypes coupled with the lack of standardization in panels of autoantibodies tested for and techniques used makes it difficult to pool data (25). While there is great interest in the use of autoantibodies to predict outcomes and survival, lack of standardization and consensus hampers clinical utilization.

## Pulmonary Disease Autoimmunity and Lung Transplant

Pulmonary disease autoimmunity and lung transplant can be seen as a ‘perfect storm’ for graft injury and rejection. While several studies have investigated autoantibodies in context of lung transplantation few have attempted to determine the clinical significance or the mechanism of action of pre-existing autoantibodies, present prior to transplantation, on post-transplant outcomes. It is important to note that many of the autoantigens identified in pulmonary disease patients are not lung-specific, but rather have a broad biodistribution. While pulmonary disease-associated autoantigens that are not lung-specific may still be clinically relevant, it is possible that lung-restricted self-antigens may also be clinically relevant with regard to LTx outcomes because they could represent biomarkers of graft health/damage or contribute pathogenically to graft damage.

**Figure 1:**
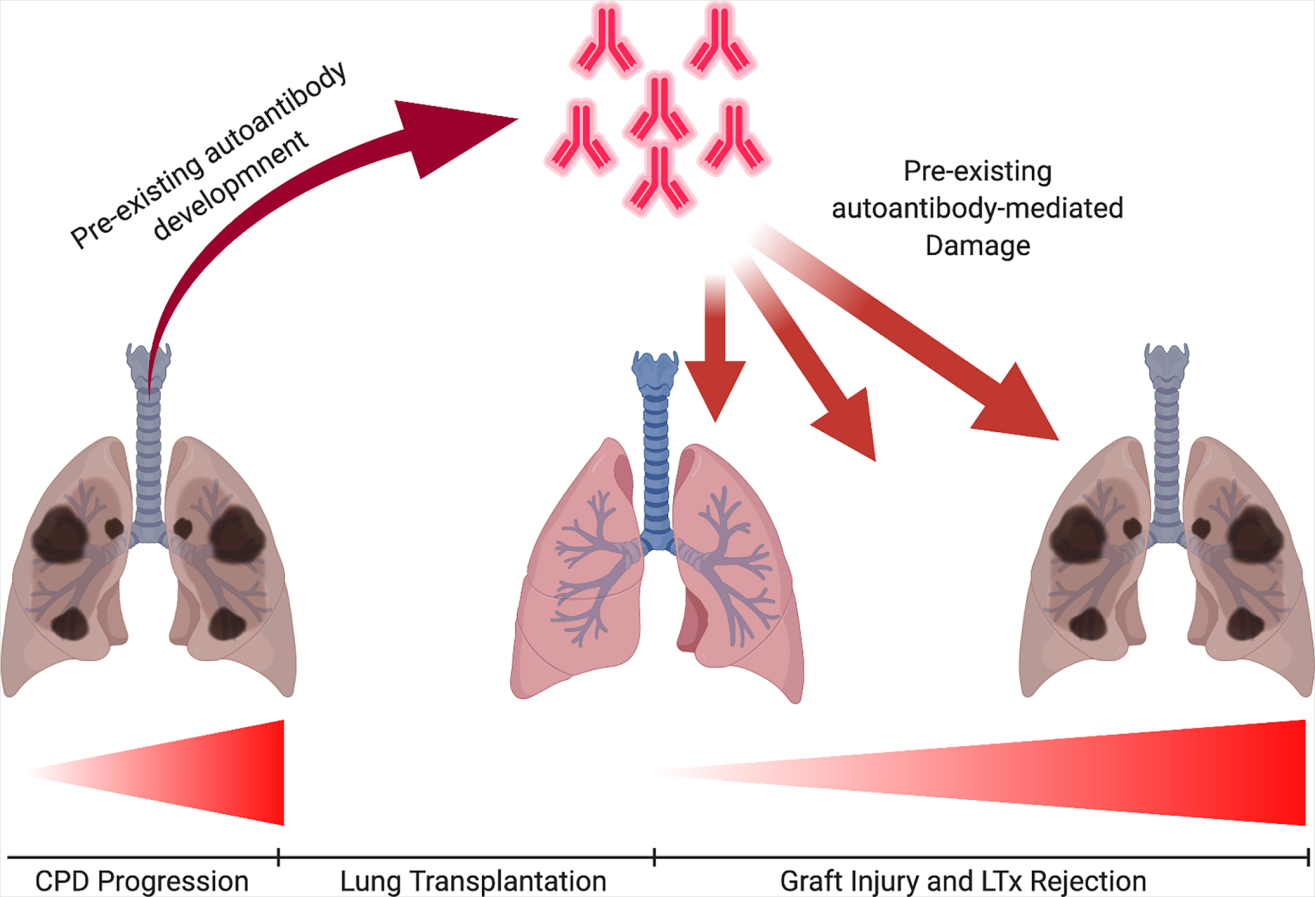
Pre-existing Autoantibody-mediated Graft Injury. Autoantibodies developed as a consequence of chronic end-stage pulmonary diseases (CPD), such COPD and ILD may pre-dispose to worse graft outcomes. Autoantibodies characteristic of COPD and ILD have been shown to not only correlate with disease severity pre-transplant but hold the potential to target the lung and induce injury post-transplantation. Pre-existing autoantibodies that have been described can promote pro-inflammatory responses *via* a variety of effector pathways, including complement activation and Fc gamma receptor mediated inflammation, the impact of which on graft rejection has not been fully explored.

In this context, pre-existing autoantibodies that recognize lung-restricted self-antigens can be produced prior to LTx (**Figure 1**). One hypothesis for their production is that natural low-affinity IgM autoantibodies recognize damaged and abnormally exposed self-proteins as antigenic and act as templates for somatic hypermutation and class switching to high-affinity IgG/IgA autoantibodies (26). Lung-restricted proteins can become antigenic through tissue destruction and inherent pulmonary disease characteristics, as previously stated. Interestingly, similar processes that expose and render self-proteins antigenic in pulmonary disease can occur to the graft. This is interesting because it could explain a mechanism by which pre-existing autoantibodies bind to and target the donor graft. For example, donor brain death induces inflammation and immune cell infiltration into the lung that potentiate cell death and tissue damage (27). Other unavoidable injuries that impact the donor organ prior to transplantation such as organ procurement strategies, cold storage, transportation, time between organ procurement to transplantation, and reperfusion of the graft have all been demonstrated to reduce viability of the lung due to severe inflammatory responses and irreversible tissue damage (28, 29). The subsequent inflammation and tissue damage drives cell death, release of ROS, and degradation of proteins thereby promoting an environment for pre-existing recipient autoantibodies present prior to transplantation to bind to damaged lung-proteins early post-transplant and induce graft injury, however, definitive evidence for this mechanism is currently lacking.

Although there are not any studies directly comparing the tissue damage that occurs in pulmonary disease to graft damage, it is reasonable to hypothesize that similar enzymatic degradation of extracellular matrix occurs early post-transplant. The innate immune cells associated with injury in both pulmonary disease and graft damage are essentially the same, and can induce post-translational modifications to ECM proteins through secretion of proteases and production of ROS. Along this line of thinking, the LTx recipient’s pre-existing autoantibodies could recognize self-antigens that are present and exposed in the new graft. Therefore, the recipient’s immune system is primed to target and attack the graft prior to transplant which could manifest as severe pre-existing autoantibody-mediated graft damage once the graft is implanted. Potential mechanisms by which pre-existing autoantibodies mediate graft damage could be increased complement activation and/or FcyR binding, which we discuss later in this review.

Although it is enticing to hypothesize about the potential pathogenicity and clinical relevance of pulmonary disease autoantibodies in LTx, definitive experimental and clinical data supporting potential mechanisms are lacking. The presence of pre-existing autoantibodies in transplant patients and their association with poor clinical outcomes has been well established in other solid organ transplants, specifically in renal and heart transplants; driving the hypothesis that pre-existing autoantibodies may also play a role in LTx outcomes (30, 31). As our understanding of autoimmune-mediated graft damage in LTx patients deepens, the association between pre-existing autoantibodies and post-LTx disorders has become an intense area of research. Identification and functional characterization of pre-existing autoantibodies in disorders such as primary graft dysfunction, chronic lung allograft dysfunction, and mortality has begun to accumulate (32–34). Below we will outline the research analyzing the association of pre-existing autoantibodies and lung transplant outcome.

### Pre-Existing Autoantibodies and Primary Graft Dysfunction

Primary graft dysfunction (PGD) is a progressive acute lung injury syndrome that manifests within the first 72 hours after lung transplantation and affects 25-30% of LTx patients (35). The ISHLT definition of PGD is based on radiographic infiltrates and P/F ratios, the ratio between arterial partial pressure and inspired oxygen, assessed at multiple time points post-LTx and is graded on a scale of Grade 0 to 3. PGD onset and grade are correlated with increased mechanical ventilation, hospital stay, cost, CLAD, and most importantly, early morbidity and mortality (36). The mechanisms responsible for PGD development are not completely understood, however, donor- and recipient-related clinical risk factors (age, gender, race, and comorbidities) and IRI have all been associated with PGD etiology [For detailed reviews see Diamond et al. (35), Suzuki et al. (37), and Altun et al. (38)].

Recently, the presence of pre-existing autoantibodies has been associated with increased risk of PGD in multiple retrospective clinical studies. The most well studied pre-existing autoantibodies correlated with PGD onset are Collagen V (Col V) and K-alpha1-tubulin (KAT). Col-V is a minor fibrillar collagen found in the lungs where it is normally sequestered from recognition by collagen I and collagen III overlays. Exposure to Col-V is hypothesized to occur upon matrix metalloprotease (MMP) degradation of the extracellular milieu during ischemia reperfusion injury, and immunogenic Col-V fragments can be found in BAL fluid (39–41). KAT is a gap junction protein that was observed to promote fibrogenic signaling after binding to airway epithelial cells (42). Clinically, anti-Col-V and anti-KAT autoantibodies have been identified in different CPD populations at different frequencies. Approximately 30% of ILD patients and 20% of COPD patients are positive for pre-existing anti-Col-V and anti-KAT autoantibodies (39). A second study also found that approximately 20% of LTx patients tested positive for pre-existing anti-Col-V autoantibodies and about 60% of those patients remained positive for anti-Col-V pre-existing autoantibodies after LTx (43).

**Figure 2:**
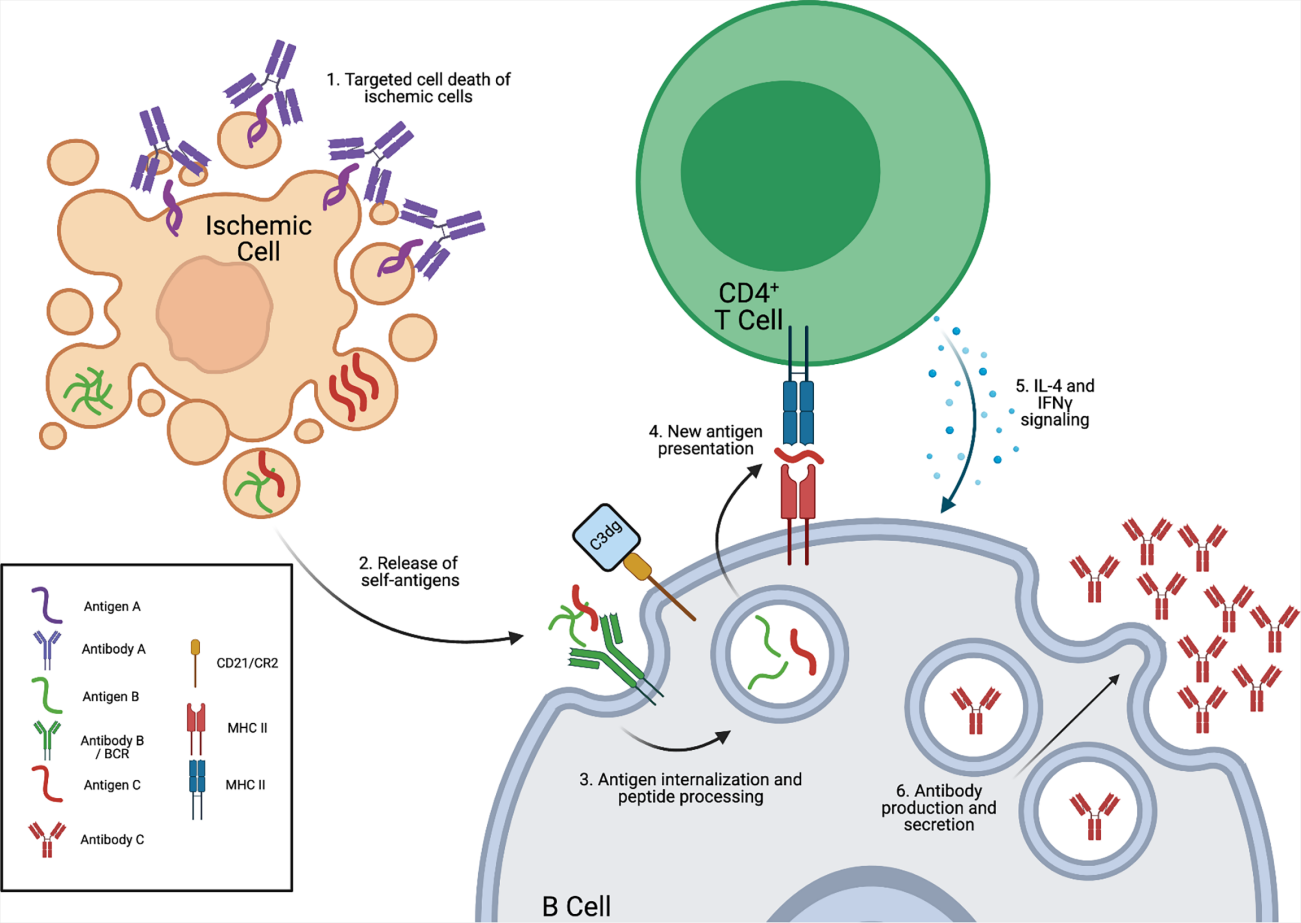
Pre-existing autoantibodies could pre-dispose to heightened epitope spreading. The existence of lung targeted autoreactive antibodies generated pre-transplantation as a consequence of the recipient’s chronic lung diseases could exacerbate cellular injury. 1. Pre-existing lung autoreactive antibodies bind within the lung upon reperfusion and induce cell injury. 2. Injury to donor lung cells releases other self-antigens. 3. Self-antigens are internalized by antigen presenting cells and presented to T cells (4) and promote B cell production of autoreactive antibodies (5 and 6).

More literature focusing on the correlation between Col-V and KAT autoantibodies and PGD, and potential mechanisms were recently reviewed by Sureshbabu et al. (32). Briefly, the presence of pre-existing anti-Col-I, anti-Col-V, and anti-KAT autoantibodies correlated with increased risk of PGD development and high levels of proinflammatory cytokines (39, 44). In a syngeneic LTx mouse model, immunization with anti-Col-V or anti-KAT resulted in dose-dependent PGD-like phenotype development. Interestingly, immunization with KAT induced an immune response that led to *de novo* anti-Col-V antibody development post transplantation, and *vice versa.* Taken together these data suggest that autoantibodies to either self-antigen are capable of inducing lung injury that leads to epitope spreading post transplantation (45). These reductive models drive the hypothesis that pre-existing lung autoantibodies could impact post-transplant outcome in those patients with diverse high titers of autoantibodies associated with their relative CPD. The presence of pre-existing recipient autoantibodies pre-transplant may not only promote more intense graft injury but further promote diverse epitope spreading, *de novo* antibody production, and an increased antibody mediated graft injury (**Figure 2**). However, human studies demonstrating pre-existing autoantibody-mediated graft injury and epitope spreading by anti-Col-V or anti-KAT have not as yet been reported.

A potential mechanism for anti-Col-V autoantibody-mediated damage can be found in elegant adoptive transfer studies performed by Iwata et al. (40). Transfer of total serum, purified serum IgG, and B cells from Col-V immunized rats into rat lung isograft recipients induced PGD-like phenotype, impaired graft function, and increased local expression of IFN-y, TNF-alpha, and IL-1ß. Interestingly, Col-V was shown to be apically expressed by epithelial cells, and when exposed to immunized rat serum, rat airway epithelial cells were sensitive to complement-mediated cytotoxicity (40). These data suggest that anti-Col-V autoantibodies promote graft damage *via* antibody-dependent complement activity and by increasing injurious pro-inflammatory cytokine production. Similarly, novel studies by Patel et al. (33) explored the impact of CPD autoantibodies on lung transplant outcomes. Using a mouse model of emphysema, the authors demonstrated that chronic exposure of mice to cigarette smoke led to the characteristic features of emphysema, such as airspace enlargement, immune cell infiltration and significantly elevated serum autoantibodies compared with non-smoke-exposed age-matched controls. To determine the impact of a full pre-existing autoantibody repertoire on lung transplant IRI the authors transplanted BALB/c donor lungs into control or chronically cigarette smoke (CS) exposed recipients. CS recipients had significantly increased lung injury and immune cell infiltration after transplant. Immunofluorescence staining revealed increased IgM, IgG, and C3d deposition in CS recipients. To exclude confounding alloreactivity and confirm the role of pre-existing autoantibodies in IRI, syngeneic Rag1 knockout transplants were performed in which recipients were reconstituted with pooled serum from CS or control mice. Serum from CS-exposed mice significantly increased IRI compared with control mice characterized by increased antibody and complement deposition. To confirm the role of autoantibodies, serum from CS was immunoglobulin depleted and adoptively transferred. Immunoglobulin depleted serum had no significant impact on transplant outcomes as compared to controls. Taken together these data demonstrate that pre-transplant CS exposure was associated with increased IgM/IgG autoantibodies, which, upon transplant, bind to the donor lung, activate complement, and exacerbate post-transplant IRI. Further studies are needed to determine, if any, the long term sequalae that a broad spectrum of autoantibodies have on graft injury, epitope spreading, and rejection. Clinical studies are also needed to corroborate these findings.

Previous literature has largely focused on Collagen V and KAT autoantibodies. As discussed earlier, and in the studies by Patel et al. (33), a diverse spectrum of autoantibodies are likely present in the lung transplant population pre-transplantation, and therefore investigating if these antibodies pre and post-transplant are clinically relevant is urgently needed. To this end, a recent large proteomic/antigen microarray study using human patient sera has successfully identified multiple different pre-existing autoantibodies that correlated with PGD. A microarray with 504 and 610 potential IgG and IgM targets, respectively, which covered 272 different proteins was created to identify new pre-existing autoantibodies. A total of 17 IgM/IgG novel autoantibodies were discovered that correlated with PGD onset (**Table 2**) (47). Another group performed a proteomic microarray analysis investigating 124 autoantigens identified 17 IgA and 3 IgG additional pre-existing autoantibodies that correlated with PGD development (See **Table 2** for list of autoantibodies) (46). Interestingly, 6 of the 17 IgA autoantibodies correlated with worse survival and those 6 autoantibodies were able to correctly classify PGD positive from PGD negative patients. Survival was not statistically different for the 3 IgG autoantibodies (Periplakin, Muscarinic acetylcholine receptor type 3 (AChR3), and Angiotensin II receptor type 1 (AT1R)) or the 11 remaining IgA autoantibodies (46). Anti-AT1R autoantibodies were the only antibodies discovered to have titers for both IgG and IgA subtypes. Although the 3 IgG and 11 remaining IgA autoantibodies do not correlate with reduced survival, they may play a role in PGD pathogenesis, but that is speculation. Anti-Periplakin IgG have been observed to decrease cell migration and induce bronchoalveolar lavage T lymphocyte proliferation in IPF, but their role in transplant is unknown (64). It is important to note that the mere presence of autoantibodies and their correlation with PGD development does not mean that they play a role in PGD pathogenesis. These autoantibodies could also represent novel predictive biomarkers for patients at risk of developing PGD.

**Table 2:** Pre-existing autoantibodies and primary graft dysfunction development.

Autoantibody	Ig Isotype	Associated with Survival	Citation
Collagen I	IgM/IgG	Yes	(44)
Collagen V	IgM/IgG	Yes	(44)
K-alpha Tubulin	IgM/IgG	Yes	(44)
Filaggrin	IgA	Yes	(46)
Factor P	IgA	Yes	(46)
Heparan sulfate	IgA	Yes	(46)
Laminin	IgA	Yes	(46)
RSV antigen	IgA	Yes	(46)
CRP antigen	IgA	Yes	(46)
Factor B	IgA	No	(46)
ERP29	IgA	No	(46)
Enolase	IgA	No	(46)
Endothelial cell extract	IgA	No	(46)
rhHSPG2	IgA	No	(46)
AGTR1	IgA/IgG	No	(46)
Proteopglycan	IgA	No	(46)
c-MYC	IgA	No	(46)
SDC1	IgA	No	(46)
Aggrecan	IgA	No	(46)
Complement C1q	IgA	No	(46)
Periplakin	IgG	No	(46)
Acetylcholine receptor (AchR3)	IgG	No	(46)
EGFR	IgM/IgG	No	(47)
MBP	IgM/IgG	No	(47)
MLANA	IgM/IgG	No	(47)
MUC1	IgM/IgG	No	(47)
MYCL1	IgM/IgG	No	(47)
PLCG1	IgM/IgG	No	(47)
PRKCA	IgM/IgG	No	(47)
HSP90AA1	IgM/IgG	No	(47)
IGF1R	IgM/IgG	No	(47)
RB1	IgM/IgG	No	(47)
CERK	IgM/IgG	No	(47)
HSPD1	IgM/IgG	No	(47)
TEP1	IgM/IgG	No	(47)
CYP3A4	IgM/IgG	No	(47)
SOCS3	IgM/IgG	No	(47)
TARP	IgM/IgG	No	(47)
TP53	IgM/IgG	No	(47)

Pre-existing autoantibodies found in COPD and ILD patients compared to healthy controls.

Anti-AT1R autoantibodies have been previously identified in kidney, heart, and liver transplants with their presence associated with poor prognosis (65, 66). Specifically, the presence of pre-Tx anti-AT1R autoantibodies was associated with fibrosis and vessel occlusion in renal transplant, suggesting a potential mechanism through which anti-AT1R could promote PGD (67). Anti-AT1R acts as an agonist on AT1R, which leads to sustained AT1R activation which is thought to promote vasoconstriction, inflammation and fibrosis (68–70). In the context of kidney Tx, anti-AT1R-mediated prolonged signaling of AT1R which induced pro-inflammatory and pro-coagulant processes *via* activation of extracellular signaling-regulated kinase 1/2 and nuclear factor-kB in endothelial and vascular smooth muscle cells (69). Other studies have purported a pathogenic role of anti-AT1R through modulation of AT1R expressing immune cells (65). AT1R expression is observed on monocytes, T and B cells, and serum from anti-AT1R positive systemic sclerosis patients induced peripheral blood mononuclear cell production of pro-inflammatory chemokines, IL-8 and CCL18 (71). Interestingly, anti-AT1R antibodies were found to belong to the complement fixing IgG1 and IgG3 subclasses, however, its mechanism of action is reportedly complement independent based on the lack of complement deposition in anti-AT1R positive patients (The impact of IgG structure and subclass on function is discussed later in this review) (69, 72, 73). Although these elegant studies proposed pathogenic mechanisms for anti-AT1R in kidney transplant and systemic sclerosis patients, to the best of our knowledge no experimental evidence exists demonstrating pathogenic functions of anti-AT1R in the context of LTx and PGD onset.

One study identified anti-AT1R in conjunction with anti-endothelin type A receptor (ETAR) in cystic fibrosis patients both pre- and post-Tx (74). A second study analyzing pre- and post-LTx sera of 162 patients from 3 centers also identified the presence of anti-AT1R and anti-ETAR. The presence of human leukocyte antigen (HLA)-specific antibodies and strong anti-AT1R and anti-ETAR binding significantly reduced freedom from *de novo* donor specific antigen (DSA) development post-LTx (75). *De novo* DSA development in LTx is well known to promote acute and chronic rejection, and reduce survival (76–78). Interestingly, the presence of anti-ETAR is highly associated with anti-AT1R in pediatric kidney transplant which may suggest another example of epitope spreading, however this has not been proven (79).

Overall, more research into pre-existing autoantibodies and PGD is needed. Large screenings, such as those used to discover autoantibodies in COPD and ILD patients, would help identify and measure pre-existing autoantibodies before and after LTx. Following identification, functional analyses of pre-existing autoantibodies are needed to determine if they are biomarkers, pathogenic, both, or neither. This information could hold the key to novel pre-Tx or perioperative treatments that reduce the risk of PGD development. Reducing the incidence of PGD is essential step to overcome poor survival. PGD is also a major risk factor for chronic lung allograft dysfunction (CLAD), the main cause of late-stage mortality (35, 80).

### Pre-Existing Autoantibodies and Chronic Lung Allograft Dysfunction

Chronic lung allograft dysfunction (CLAD) remains the largest obstacle to long term allograft survival. ISHLT defines CLAD as the consistent decline in forced expiratory volume in 1 second (FEV_1_) from baseline FEV_1_ following LTx. There are three CLAD phenotypes referred to as bronchiolitis obliterans syndrome (BOS), restrictive allograft syndrome (RAS, previously known as restrictive CLAD), and neutrophilic reversible allograft dysfunction (NRAD). However, NRAD was not included in the most recent ISHLT consensus report. BOS, the most common CLAD phenotype, accounts for approximately 60-75% of CLAD cases (81) [Extensive reviews of CLAD can be found in Verleden et al. (82) and Glanville et al. (83)].

To date, three studies have evaluated whether pre-existing autoantibodies are associated with CLAD development. In the first, patients with pre-existing anti-Col-V and anti-KAT autoantibodies were at increased risk for development of HLA-antibodies and for BOS development (44). In the second, a single center study investigated the presence of pre-existing autoantibodies in ‘stable BOS’ and ‘progressive BOS’ patients. Using a proteomic microarray with 124 self-antigens, analysis revealed 16 IgG pre-existing autoantibodies that were elevated in the progressive BOS patients as compared to stable BOS patients (See **Table 3** for list of autoantibodies). A subset of 6 out of the 16 pre-existing autoantibodies correlated with worse BOS free survival. Those 6 IgG autoantibodies remained elevated three months and one-year post-Tx. Another microarray investigating 751 different antigens investigated autoantibodies in a subset of LTx patients with no-to-mild BOS compared to moderate-to-severe BOS patients (84). Approximately 28 pre-existing autoantibodies were elevated in the moderate-to-severe BOS patient population compared to the no-to-mild BOS patient population (**Table 3**). Interestingly, 6 of the IgM/IgG autoantibodies identified were also elevated in a subset of patients that develop PGD, suggesting a connection between PGD and BOS development (47, 84). However, further studies are required to determine whether these pre-existing autoantibodies are pathogenic or epiphenomena of chronic rejection (85).

**Table 3:** Pre-existing autoantibodies and chronic lung allograft dysfunction.

Autoantibody	Ig Isotype	CLAD Phenotype	Associated with Survival	Citation
Collagen V	IgM/IgG	BOS	Yes	(39–41)
K-alpha Tubulin	IgM/IgG	BOS	Yes	(42, 136)
ALDOC	IgG	BOS	No	(85)
Aldolase muscle	IgG	BOS	No	(85)
APEX1	IgG	BOS	Yes	(85)
B7H4	IgG	BOS	Yes	(85)
BAFF	IgG	BOS	No	(85)
BPI	IgG	BOS	No	(85)
Complement C1q	IgG	BOS	No	(85)
Complement C6	IgG	BOS	No	(85)
Fuca1	IgG	BOS	No	(85)
Clycyl tRNA synthetase EJ	IgG	BOS	No	(85)
MAGEA3	IgG	BOS	No	(85)
MBP	IgG	BOS	Yes	(85)
Nup62	IgG	BOS	Yes	(85)
Ro/SSA(52/60 Kda)	IgG	BOS	No	(85)
Troponin I	IgG	BOS	No	(85)
Troponin I T C Terniary Complex	IgG	BOS	No	(85)
NTF3	IgM/IgG	BOS	No	(84)
CCl5	IgM/IgG	BOS	No	(84)
NPPB	IgM/IgG	BOS	No	(84)
TBX21	IgM/IgG	BOS	No	(84)
PRKCA	IgM/IgG	BOS	No	(84)
JUN	IgM/IgG	BOS	No	(84)
GATA3	IgM/IgG	BOS	No	(84)
NPPA	IgM/IgG	BOS	No	(84)
GCG	IgM/IgG	BOS	No	(84)
CXCL10	IgM/IgG	BOS	No	(84)
IL-11	IgM/IgG	BOS	No	(84)
PLD3	IgM/IgG	BOS	No	(84)
SSB	IgM/IgG	BOS	No	(84)
IGF1R	IgM/IgG	BOS	No	(84)
CNTNAP1	IgM/IgG	BOS	No	(84)
HPSD1	IgM/IgG	BOS	No	(84)
FOXP3	IgM/IgG	BOS	No	(84)
CRYBB1	IgM/IgG	BOS	No	(84)
TNF	IgM/IgG	BOS	No	(84)
TP53	IgM/IgG	BOS	No	(84)
CASP3	IgM/IgG	BOS	No	(84)
TARP	IgM/IgG	BOS	No	(84)
CYP3A43	IgM/IgG	BOS	No	(84)
GAD2	IgM/IgG	BOS	No	(84)
CASP8	IgM/IgG	BOS	No	(84)
HSP90AA1	IgM/IgG	BOS	No	(84)
EEF1A1	IgM/IgG	BOS	No	(84)
SNCG	IgM/IgG	BOS	No	(84)
HSPD1	IgM/IgG	BOS	No	(84)

Pre-existing autoantibodies associated with CLAD compared to control (Col-V and KAT) and pre-existing autoantibodies associated with progressive CLAD compared to stable CLAD (85).

There are likely more pre-existing autoantibodies associated with CLAD that need to be identified. Interestingly, some of the same autoantibodies identified in the Kaza et al. (85) study have been described and associated with other solid organ transplants (30, 86–88). For example, anti-AGTR1, anti-LG3, and anti-vimentin pre-transplant autoantibodies have been correlated with high risk of graft failure in kidney and heart transplant (30, 86–88). The presence of anti-vimentin autoantibodies in patients awaiting solid organ transplantation (heart and lung transplantations) was previously observed to be low (approximately 6%) (89). Alternatively, a second study identified that circulating vimentin and anti-vimentin IgG autoantibodies were increased in IPF patients compared to healthy individuals (90). Interestingly, these data demonstrate that anti-vimentin autoantibodies have been identified in ILD patients and vimentin expression increases following LTx. However, any association between anti-vimentin autoantibodies and LTx outcome has not been studied (91). The list of potential pre-existing autoantibodies associated with CLAD is extensive based on the sheer number of different autoantibodies identified in COPD and ILD patients, and more research is needed to streamline and elucidate their clinical significance in post-LTx outcome (12, 25).

## Potential Mechanisms of Graft Injury by Pre-Existing Autoantibodies

In addition to gaining a better understanding of the epitope targets, the functional characterization of pre-existing autoantibodies in transplantation is understudied. There is support and agreement that pre-existing autoantibodies are associated with graft injury and poor prognosis, however, conclusive evidence suggesting whether they are biomarkers of injury, pathogenic, beneficial, or simply epiphenomena of the diseases are lacking. Teasing out the biological function of pre-existing autoantibodies may be difficult due to the interconnectedness of antibodies in multiple different immune responses (i.e., complement fixing and FcyR interactions) and the heterogeneity of antibody characteristics (i.e., Ig class and class subtypes). Nonetheless, functional characterization could lead to the development of patient risk stratification strategies if the autoantibodies prove to be biomarkers. Further, a better understanding of the type and function of autoantibodies could be leveraged to develop novel antibody-targeted therapeutics designed to reduce graft damage if autoantibodies are found to be pathogenic.

### Pre-Existing Autoantibody Immunoglobulin Classes, Subtypes, and Fab Specificity

The inherent heterogeneity among antibodies and their structure gives them the ability to mediate different immune responses. Antibodies, or immunoglobulins, are composed of two heavy and two light chains. The heavy chain, or constant domain (Fc), specifies effector function and the light chain, or variable domain (Fab), binds to antigen. There are five main Fc immunoglobulin (Ig) isotypes; IgM, IgG, IgA, IgE, and IgD. The IgG and IgA isotypes can be split into IgG1, IgG2, IgG3, and IgG4, and IgA1 and IgA2 subclasses, respectively.

The Fc immunoglobulin classes differ in various properties such as structure, complement fixation, and FcyR binding [For detailed review see: Schroeder and Cavacini (92)]. Historically, little attention was paid to IgA, IgD, and IgE in the context of autoantibodies because they function predominantly in mucosal response to pathogens, homeostasis, and allergens, respectively, and do not interact with FcyRs, and are incapable of complement fixation. Furthermore, while IgM makes up a group of antibodies termed natural antibodies that are capable of binding autoantigens, natural IgM antibodies have relatively low affinity and are not hypothesized to be drivers of autoimmune disease or pathogenesis (92). Natural IgM antibodies role in IRI and IgG (total IgG, not IgG subtypes) have received most of the attention from those studying autoantibodies (93–98). While focusing on total IgG autoantibodies in pulmonary disease has proven to be fruitful, we know little about the role if any of other Ig classes and IgG subtypes in autoreactive-mediated graft damage. Ig isotypes and subclasses have been studied in the context of alloantibodies (DSA and anti-HLA antibodies), however much less is known with regard to pre-existing autoantibodies (non-HLA autoantibodies). Comparatively fewer reports have investigated the mechanistic significance of Ig sub-classes of pre-existing autoantibodies in LTx. Elucidating whether Ig subclasses are clinically relevant in the context of LTx may well provide novel information that could be therapeutically leveraged to improve graft outcomes (33). Therefore, it is essential to study the multiple characteristics of pre-existing autoantibodies to comprehensively understand if they have any impact on graft injury. Below we will discuss different immunoglobulins and postulate on the potential impact they may have on pre-existing autoantibody-mediated graft damage.

*IgA*. IgA has been well studied in the context of mucosal immunity and renal function (IgA nephropathy), however, little is known about IgA and solid organ transplantation. A singular report has analyzed pre-existing IgA autoantibodies in LTx patients. Kaza et al. (46) performed a proteomic microarray of 124 potential antigens to measure serum profiles of IgG and IgA pre-existing autoantibodies. Although IgG is present at a higher concentration in the serum than IgA, the authors reported larger numbers of pre-existing IgA autoantibodies compared to pre-existing IgG autoantibodies. Furthermore, only pre-existing IgA autoantibodies correlated with PGD development and survival (46). One explanation for higher IgA pre-existing autoantibodies could be the fact that IgA plays an important role in mucosal immunologic defense. Some of the IgA pre-existing autoantibodies identified were directed against proteoglycans that may be present in the respiratory mucosal system and extracellular matrix. IgA can promote pro-inflammatory responses when interacting with the FcαRI on neutrophils (99–101). Given the importance of the respiratory mucosal system, neutrophil infiltration and damage in LTx, it is reasonable to hypothesize that IgA-immune complexes may promote graft damage after binding mucosal and ECM autoantigens. Interestingly, recent evidence suggests that molecular phenotyping of mucosal biopsies can be used to detect and accurately diagnose rejection following LTx (102).

A group of studies examining pre-existing IgA autoantibodies, although in kidney Tx patients, identified a positive correlation with kidney graft survival. In contrast to most examples thus far, the authors demonstrated that the presence of pre-existing IgA autoantibodies directed against the Fab region of human IgG molecule increased the chance of kidney graft survival (103). Although the aforementioned studies were in a different solid organ Tx system, these studies suggest that whether an autoantibody is pathogenic or advantageous is dependent on the antigen. More support for IgA antibodies impacting transplant outcomes lies in the observation that low pre-transplant levels of systemic IgA antibodies have been associated with increased post-LTx mortality (104). Although no specific antigens were investigated in this study, increased mortality was hypothesized to result from increased risk of post-LTx infection, therefore, not necessarily autoantibody mediated. Nonetheless, these studies demonstrate that much more research is needed to better understand the relationship between IgA antibodies, their corresponding antigens, differences in IgA subclass, and their function/role in transplant outcome.

**Figure 3:**
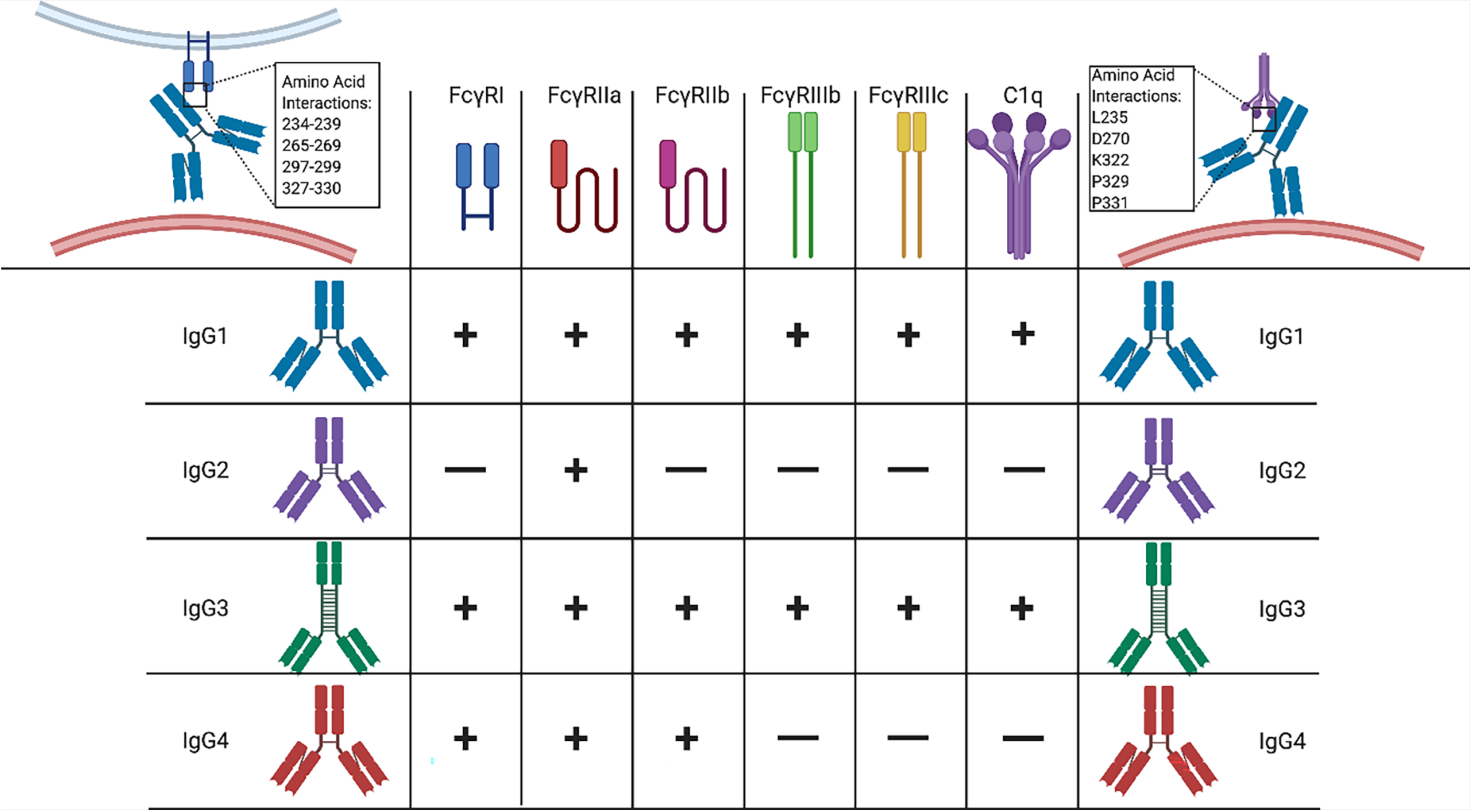
Pre-Existing Autoantibody Characteristics Impact Effector Functions. Autoantibodies bind to a variety of different antigens expressed on the cell surface of damaged cells or damaged/modified proteins that exist in the extracellular space. Upon binding, autoantibodies work to fine-tune the activation or suppression of the immune response. Inherent antibody characteristics such as antigen specificity, Ig class, IgG/A subclass, and glycosylation give autoantibodies the ability to regulate the immunological response to stimuli. Antigenic specificity is determined by the Fab’ portion of the antibody while effector function is dependent on the Fc portion of the antibody. Immune responses can be pro-inflammatory or anti-inflammatory based on the target antigen. Ig classes (IgM/G/A/E/D) have different immune activation capabilities and activate different immune pathways. For example, IgG and IgM are able to fix complement while IgA, IgD, and IgE are not. IgG/A subclasses are also capable of modulating effector functions. IgG1-4 have different complement fixation capabilities and interact with different FcyR’s, thereby, fine-tuning the immune response.

*IgG.* The connection between IgG and transplant success has been well studied for over forty years. IgG has been studied in the context of DSA and HLA- and non-HLA antibodies, antibody mediated rejection, and hypogammaglobulinemia, or immunoglobulin deficiency, in lung transplant. In general, studies examining total IgG indicate that expression levels and their antigen of target (HLA and non-HLA) play crucial roles in transplantation outcome. However, significantly less research has focused on the influence and importance of different IgG subclasses. The four IgG subclasses, IgG1, IgG2, IgG3, and IgG4, have different physical properties, abundance, and effector functions. For example, IgG1-IgG3 are complement fixing with IgG3 having highest binding efficiency to C1q followed by IgG1 and IgG2 (105). However, IgG4 does not appear to have any complement activating ability and is often associated with immunotolerance (106, 107). IgG subclasses also demonstrate different binding affinities for FcyRs: IgG1 and IgG3 bind FcyRI-III, while IgG4 binds FcyR I and II, and IgG2 only binds FcyRII (92). More details describing the differences in IgG subclasses can be found in elegant reviews by Schroeder and Cavacini (92) and in **Figure 3**. Overall, the differences in these properties between subclasses could greatly influence the effector functions of a pre-existing IgG autoantibody.

Unfortunately, very few papers have delved into characterizing the IgG subclasses of pre-existing autoantibodies in LTx. Our group recently performed a study measuring the level of anti-elastin pre-existing autoantibody IgG subclasses in an emphysema mouse model. We discovered that all four IgG anti-elastin pre-existing autoantibodies are elevated in CS exposed mice compared to controls. These pre-existing autoantibodies were determined to be pathogenic, however, it remains unclear whether one subclass of IgG is more pathogenic than another (33). The hypothesized mechanism of action for pre-existing autoantibody-mediated damage includes complement fixation and activation, therefore, IgG3 and IgG1 would likely be the predominantly pathogenic IgG subclasses in this case, but further studies are needed.

Due to the lack of publications on pre-existing IgG subclass autoantibodies, support for this hypothesis can be found by investigating the role of IgG subclasses in donor specific antigen DSA HLA-antibodies. For example, a retrospective study analyzing individual IgG subclass DSAs following liver transplant found that patients positive for DSAs presented with IgG1 most often, followed by IgG3, and then IgG4 and IgG2. Patients that demonstrated chronic rejection often times had a combination of IgG subclasses, but patients with IgG3 DSAs were at increased risk of graft loss (108). A similar observation was made in patients following renal transplant. Antibody mediated rejection was more prevalent in patients with IgG1/IgG3 *de novo* DSA antibodies compared to a mixture of complement and non-complement fixing *de novo* DSA antibodies (109). In contrast, patients with pre-existing complement-fixing DSA IgG subclasses did not demonstrate significantly different incidence of antibody mediated rejection (AMR) as compared to patients with a combination of pre-existing IgG1/IgG3 and IgG2/IgG4 DSA antibodies in kidney transplant (110). However, there was a trend that ABMR was higher 6 months post-Tx in patients with pre-existing complement fixing DSA antibodies. Taken together, these data suggest that IgG subclass DSA antibodies, whether pre-existing or developed *de novo*, impact outcomes. Therefore, it is reasonable to hypothesize that pre-existing non-HLA autoantibody Ig subtype analysis could prove beneficial in predicting graft outcomes.

*IgE/IgD.* To date, there are no studies investigating IgE or IgD autoantibodies in the context of transplantation.

### Pre-Existing Autoantibody Glycan Profiles

**Figure 4:**
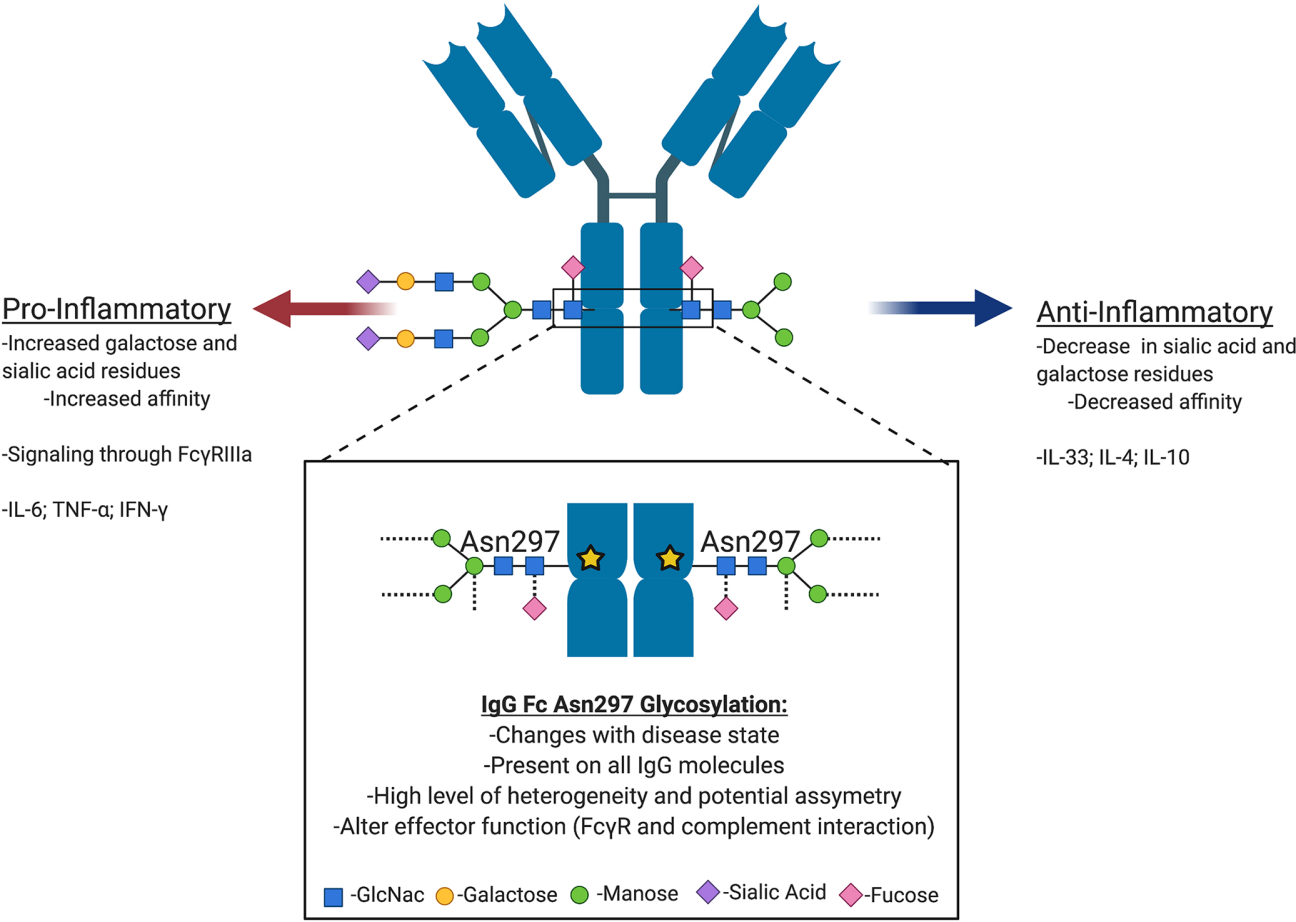
Antibody N-glycosylation can influence antibody effector functions. Antibodies are asymmetrically glycosylated on a conserved Asn297 residue within each of their Fc fragments. There are estimated to be at least 33 different glycosylation signatures that can be post-translationally added to an antibody. The fact that each antibody can be asymmetrically glycosylated on each Fc fragment suggests that there are many iterations of glycan modifications. Antigen-Antibody complexes can thus stimulate pro- or anti-inflammatory responses based on the combination of glycans added.

Antibody glycosylation is a fundamental biochemical reaction determined by various biological factors that significantly influence disease and immune states (111–121). All Ig families are glycosylated and previous review articles have elegantly highlighted the biological significance of Ig glycosylation in multiple disease settings (113, 114, 120, 121). A conserved site of N-glycosylation at N297 in the heavy chain (HC) region is common in the IgG class of antibodies which maintains the structure and stability of the Fc region (**Figure 4**) (92). IgG glycan alterations have significant impact on effector functions, such as immune cell FcyR interactions and altered complement activation, with some glycan signatures being predominantly pro-inflammatory, while others render IgG anti-inflammatory (120, 122–128). Given the importance of Ig in transplantation, it is clear that investigating Ig characteristics such as glycosylation state could well be essential in increasing our understanding of the role of antibodies in solid organ transplant. Unfortunately, to date little is known regarding Ig glycosylation status in solid organ transplant in general, and even less is known regarding autoantibodies in LTx.

Experimental evidence has demonstrated that serum and IgG N-glycan signatures change with disease and impact disease progression [Reviewed by: Goulabchand et al. (112); Maverakis et al. (121); and Zhou et al. (129)]. For example, COPD patients have more complex glycan structures and a decrease in monogalactosylated species compared to healthy individuals (130). In fact, the more complex glycan structures correlate with COPD GOLD stage progression. Similar serum N-glycan signature changes were observed in the IgG N-glycan signature (130). Therefore, it would be reasonable to hypothesize that LTx patients present with pre-existing autoantibodies that have pro-inflammatory N-glycan signatures.

In support of this hypothesis, what little data that does exist on glycosylation in Tx has demonstrated that changes in the N-glycome are predictive of poorer outcomes in liver and kidney transplantation (131–134). Interestingly, one group studying IgG N-glycosylation in hematopoietic stem cell transplantation (HSCT) found that the recipients’ IgG glycosylation status post-Tx does not mimic the donor profile, and instead is determined by environmental factors of the host (135). These data could suggest that the IgG N-glycan profile present pre-transplant could be pro-inflammatory due to underlying pulmonary disease. A well-established pro-inflammatory environment resulting from pulmonary disease combined with pro-inflammatory processes inherent to transplantation may lead to environmental pressures that maintain pro-inflammatory IgG N-glycosylation states. Furthermore, unlike in HSCT, the LTx recipient’s B cell population would remain of recipient origin and therefore already be primed to produce pro-inflammatory N-glycan signature IgG autoantibodies.

## Concluding Remarks

Current immunosuppressive strategies only start upon transplantation and have little impact on B cell functions and pre-existing antibodies. While de-sensitization strategies exist for pre-sensitized patients no studies have investigated the impact of autoantibody depletion on lung transplant outcomes. Pre-existing autoantibodies such as anti-Col-V, anti-KAT, and anti-elastin have been demonstrated to cause inflammatory-mediated graft damage and promote epitope spreading in murine models. Furthermore, anti-Col-V and anti-KAT autoantibodies have been identified in patient samples and correlate with LTx rejection. Multiple other pre-existing autoantibodies have been inconsistently correlated with increased risk of PGD, and CLAD. While a greater understanding of the repertoires present is slowly emerging, little is still known regarding the antibody characteristics that may fine-tune the pathogenic mechanisms of autoantibodies in organ transplantation. Immunoglobulin class (IgM, IgG, IgA, IgE, IgD) and subclass (IgG1-4; IgA1-2) have all been shown to interact with different Fc receptors and have different complement fixation capabilities. Structural changes to the Fc portion of immunoglobulins such as glycosylation can also greatly impact the antibody-mediated inflammatory response. Unfortunately, little is known with regard to these antibody features and as such the pathogenic mechanisms and corresponding antibody characteristics of disease specific pre-existing autoantibodies is largely still unknown. Gaining a better understanding of the epitopes, Ig-sub class and glycosylation state may facilitate the development of novel personalized pharmacotherapeutics that can be leveraged to augment immunosuppression and improve the outcomes of lung transplant recipients.

## Author Contributions

All authors contributed to the conceptualization, writing, and editing of the manuscript. All authors contributed to the article and approved the submitted version.

## Funding

These studies were supported by grants from the NIH (NHLBI R1R01HL 140470-01A1, NHLBI 3RO1HL140470-03S1 **an**d NIAID U01 AI132894-01 to CA), TL1 TR001451 to AM, U01CA242096 to PMA (multi-PI), (NHLBI K24-HL115354, NHLBI R01HL087115, and NHLBI U01-HL145435 to JC).

## Conflict of Interest

The authors declare that the research was conducted in the absence of any commercial or financial relationships that could be construed as a potential conflict of interest.

## Publisher’s Note

All claims expressed in this article are solely those of the authors and do not necessarily represent those of their affiliated organizations, or those of the publisher, the editors and the reviewers. Any product that may be evaluated in this article, or claim that may be made by its manufacturer, is not guaranteed or endorsed by the publisher.
